# Heart Rate Variability and Pregnancy Complications: Systematic Review

**DOI:** 10.2196/44430

**Published:** 2023-06-05

**Authors:** Zahra Sharifi-Heris, Amir M Rahmani, Anna Axelin, Mahkameh Rasouli, Miriam Bender

**Affiliations:** 1 University of California Irvine Irvine, CA United States; 2 University of Turku Turku Finland

**Keywords:** autonomic nervous system, heart rate variability, pregnancy complication, pregnancy, maternal, hypertensive disorder, fetal growth, global developmental delay, hypertension

## Abstract

**Background:**

The autonomic nervous system (ANS) is known as a critical regulatory system for pregnancy-induced adaptations. If it fails to function, life-threatening pregnancy complications could occur. Hence, understanding and monitoring the underlying mechanism of action for these complications are necessary.

**Objective:**

We aimed to systematically review the literature concerned with the associations between heart rate variability (HRV), as an ANS biomarker, and pregnancy complications.

**Methods:**

We performed a comprehensive search in the PubMed, Medline Completion, CINAHL Completion, Web of Science Core Collection Classic, Cochrane Library, and SCOPUS databases in February 2022 with no time span limitation. We included studies concerned with the association between any pregnancy complications and HRV, with or without a control group. The PRISMA (Preferred Reporting Items for Systematic Reviews and Meta-Analyses) guideline was used for the review of the studies, and Covidence software was used for the study selection process. For data synthesis, we used the guideline by Popay et al.

**Results:**

Finally, 12 studies with 6656 participants were included. Despite the methodological divergency that hindered a comprehensive comparison, our findings suggest that ANS is linked with some common pregnancy complications including fetal growth. However, existing studies do not support an association between ANS and gestational diabetes mellitus. Studies that linked pulmonary and central nervous system disorders with ANS function did not provide enough evidence to draw conclusions.

**Conclusions:**

This review highlights the importance of understanding and monitoring the underlying mechanism of ANS in pregnancy-induced adaptations and the need for further research with robust methodology in this area.

## Introduction

### Background

Various physiological changes occur during pregnancy that contribute to optimal growth and development of the fetus and help protect the mother from pregnancy and delivery complications [1]. These changes are regulated through a nonlinear complex relationship between various vital systems in the body. The autonomic nervous system (ANS) is known as a critical regulatory system for pregnancy-induced adaptations [2]. The importance of the ANS lies, to a great extent, in the fact that every organ of the human body is innervated and thus regulated by the ANS [3,4]. This systemic innervation enables the ANS to restore relationships between the individual functional systems after a disturbance of the balance of the human body with the help of certain adjusting reactions [5]. Sympathetic nervous system (SNS) and parasympathetic nervous system (PNS) components, as 2 functional elements of ANS, have complementary roles to mediate the hemodynamic adaptation in the body. The SNS directs the body's rapid involuntary response to various internal or external demands [6] and mediates the vigilance, arousal, and activation of the bodily responses to adapt to increased metabolic needs in response to internal and external stimuli including pregnancy [7]. The PNS, on the other hand, is responsible for stimulation of “rest-and-digest” or “feed-and-breed” activities that occur when the body is at rest [8].

During pregnancy, hemodynamic adjustment is one of the main adaptations regulated by the ANS. This adaptation includes adjusting blood pressure, blood volume, and heart activity. Systemic vasodilation is the primary initial hemodynamic event that begins during the luteal phase of the menstrual cycle following the release of chemical mediators by the corpus luteum and is further amplified by adjunct factors including placental hormones and the vasodilatory sex steroid estrogen that are present during pregnancy [9-11]. The outcome of systemic vasodilation is a series of systemic accommodations regulated by the ANS that adapt to the pregnancy demands known as “hemostasis” but is dynamic and complex in function [12]. One of the initial hemodynamic accommodations is an increased volume of circulating blood (plasma, red cells), resulting in 40% to 45% higher volume than prior to pregnancy, in response to systemic vasodilation [13]. Due to the increased blood volume and decreased vascular resistance, heart rate and cardiac output increase, but maternal blood pressure is not elevated. These hemodynamic adaptations provide the required blood for the fetal need for growth [13] through increased sympathetic tone and parasympathetic withdrawal.

Heart rate variability (HRV) is a well-known, noninvasive variable that has been commonly used in the recent literature to detect various physical, and psychological disorders resulting from ANS dysfunction [14]. HRV, by definition, is the variation in the beat-to-beat (RR or NN) interval and measures oscillations in the interval between consecutive heart beats and reflects the variability in the intervals between R waves [15]. By function, HRV is representative of interrelated regulatory systems that indicate the person’s adaptation to internal and external stressors. Optimal variability indicates the responsiveness of the ANS and sympathetic-parasympathetic components to deal with the stressor [15]. Variability in the heart rate indicates the flexibility to cope with the uncertain and changing environment through the cardiovascular system. HRV is a surrogate parameter of the ANS reflecting the complex interaction between organ systems, and specifically the brain and cardiovascular system, to maintain hemostasis [16].

HRV is interpreted by various mathematical computations on the interbeat interval. These include time domain (eg, standard deviation of the normal-to-normal R-R intervals [SDNN], root mean square of successive differences between normal heartbeats [RMSSD]), frequency domain (eg, low frequency [LF], high frequency [HF], LF/HF, very low frequency [VLF]), and nonlinear (eg, entropy) metrics for which the SNS, PNS, or both may contribute. The PNS mainly contributes to HF, VLF, and RMSSD, and both the SNS and PNS play a role in LF, LF/HF, and SDNN (see Multimedia Appendix 1) [15].

Literature that has used HRV as an ANS biomarker has suggested that ANS activity is shifted toward higher sympathetic and lower vagal modulation in response to pregnancy-induced demands over the course of a pregnancy [17]. However, methodological gaps such as noncontinuous assessment of HRV has hindered the understanding of where, when, and how these ANS alterations occur during pregnancy and whether these changes predict good or bad outcomes.

A failure in ANS regulation has been described in multiple and diverse diseases, both those that directly affect the nervous system and those affecting other organs, where they indirectly trigger or enhance pathological symptoms in the body [18]. It has been suggested that there is absolutely no disorder nor ailment in which the ANS plays no role [5]. Likewise, in pregnant individuals, ANS dysfunction has been considered one of the main contributors to the development of some maternal or neonatal disorders. Understanding the relationship between the ANS and pregnancy complications may be a pathway to investigate mechanisms of action for these life-threatening complications. This is particularly important as the growing availability of technology enables us to continuously and cost-effectively assess ANS function and the potential associated complications.

### Objectives

In this study, we aimed to systematically review the potential pregnancy complications associated with ANS function and reflected in HRV.

## Methods

### Design

We performed a systematic review using the PRISMA (Preferred Reporting Items for Systematic Reviews and Meta-Analyses) standards [19] to guide the study.

### Eligibility Criteria

The PECO (Population, Exposure, Comparison or Controls, and Outcome) framework was applied to develop the research question and inclusion criteria. Regarding population (P), all pregnant individuals were included. The exposure (E) component, which is required for all the studies, included any pregnancy-related complications during pregnancy. For the comparison group (C), studies with or without a control group were eligible to be included. Regarding outcome (O), HRV, assessed at least once during pregnancy, was considered the expected outcome for all the studies. Studies were included if they were in the English language. The exclusion criteria included articles that were a systematic review, protocol, conference, letter to the editor, unpublished or under review, or dissertation proposal.

### Information Sources

The PubMed, Medline Completion, CINAHL Completion, Web of Science Core Collection Classic, Cochrane Library, and SCOPUS databases were searched initially in February 2022 (with no time span limitation). Although we used no limitation for the time span during the search, the time span varied depending on the history of each database. See Multimedia Appendix 2 for more details. To access further studies, the reference lists of the reviewed articles and Google Scholar were checked.

### Search Strategy

Key words including “heart rate variability” and “pregnancy complications” were used for both simple and advanced searches in each database separately (see Multimedia Appendix 2 for all the terms).

### Selection Process

Selected articles were peer reviewed in the online Covidence software by 2 independent reviewers. To assess relevancy, all the studies were screened by both reviewers, ZS and MR, based on titles, abstracts, and full texts in 2 steps. In the first step, the abstracts of all the articles that were gathered from the databases were screened in terms of their relevance to our study aim. Next, articles with relevant titles or abstracts that resulted from the first step underwent a full-text assessment. To resolve any disagreement, a third reviewer, MB, was consulted.

### Data Items

We assessed results for HRV, as the main outcome collected; assessment tools used for HRV; HRV component(s); frequency and duration of the assessment; and gestational age at the assessment.

### Effect Measures

Effect measures for the studies were included if they were reported, and the significance of the difference varied from study to study.

### Quality and Risk of Bias Assessment

**Figure 1:**
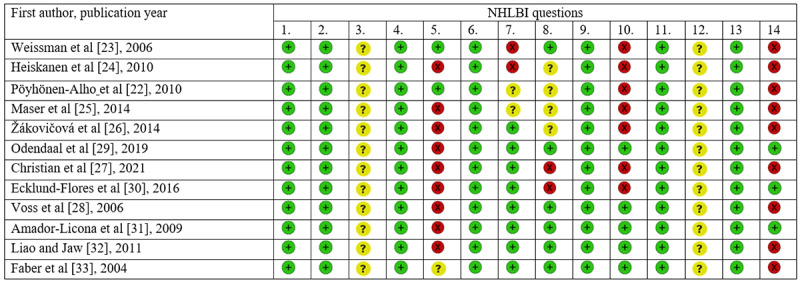
National Heart, Lung, and Blood Institute (NHLBI) quality assessment of the included studies: Question 1: Was the research question or objective in this paper clearly stated? Question 2: Was the study population clearly specified and defined? Question 3: Was the participation rate of eligible persons at least 50%? Question 4: Were all the subjects selected or recruited from the same or similar populations (including the same time period)? Were inclusion and exclusion criteria for being in the study prespecified and applied uniformly to all participants? Question 5: Was a sample size justification, power description, or variance and effect estimates provided? Question 6: For the analyses in this paper, were the exposure(s) of interest measured prior to the outcome(s) being measured? Question 7: Was the timeframe sufficient so that one could reasonably expect to see an association between exposure and outcome if it existed? Question 8: For exposures that can vary in amount or level, did the study examine different levels of the exposure as related to the outcome (eg, categories of exposure, or exposure measured as continuous variable)? Question 9: Were the exposure measures (independent variables) clearly defined, valid, reliable, and implemented consistently across all study participants? Question 9: Question 10: Was the exposure(s) assessed more than once over time? Question 11: Were the outcome measures (dependent variables) clearly defined, valid, reliable, and implemented consistently across all study participants? Question 12: Were the outcome assessors blinded to the exposure status of participants? Question 13: Was loss to follow-up after baseline 20% or less? Question 14: Were key potential confounding variables measured and adjusted statistically for their impact on the relationship between exposure(s) and outcome(s)? Green +: “yes”; red x: “no”; yellow ?: “cannot determine, not applicable, or not reported.”.

Two independent reviewers, ZS and MR, assessed the methodological quality of the selected studies using the National Heart, Lung, and Blood Institute (NHLBI) quality assessment scale for observational cohort and cross-sectional studies [20]. The NHLBI quality assessment tool, using 14 questions, assesses studies in terms of criteria such as study objectives, study population, sample size, exposures and outcome measures, and key potential confounding variables. Each study was assessed for risk of bias using “yes,” “no,” and “cannot determine/not applicable/not reported” answers for every single criterion. See Figure 1 for more details.

### Data Extraction and Synthesis

The reviewed studies were not homogenous in terms of the assessment time frame, component, and frequency; thus, a meta-analysis was not possible. A narrative synthesis was chosen to bring together the broad knowledge from a variety of approaches. This type of synthesis is not the same as a narrative description that accompanies many reviews. To synthesize the literature, we used a guideline from Popay et al [21]. The steps included (1) preliminary analysis, (2) exploration of relationships, and (3) assessment of the robustness of the synthesis. Theory development was not performed due to the exploratory nature of the research synthesized. The main synthesis consisted of extracting the descriptive characteristics of the included studies and presenting and producing a textual summary of the results. We categorized the studies based on the complication type in 4 groups. We then performed thematic analysis to extract main themes for each complication in all studies. The 4 themes developed in the results represent the main areas of knowledge available about HRV in pregnancy complications. These included study population (characteristics, sample size, exclusion criteria), design (study design, assessment duration and frequency, considered HRV metrics), measures (device used for HRV assessment), and findings.

## Results

### Study Selection

**Figure 2:**
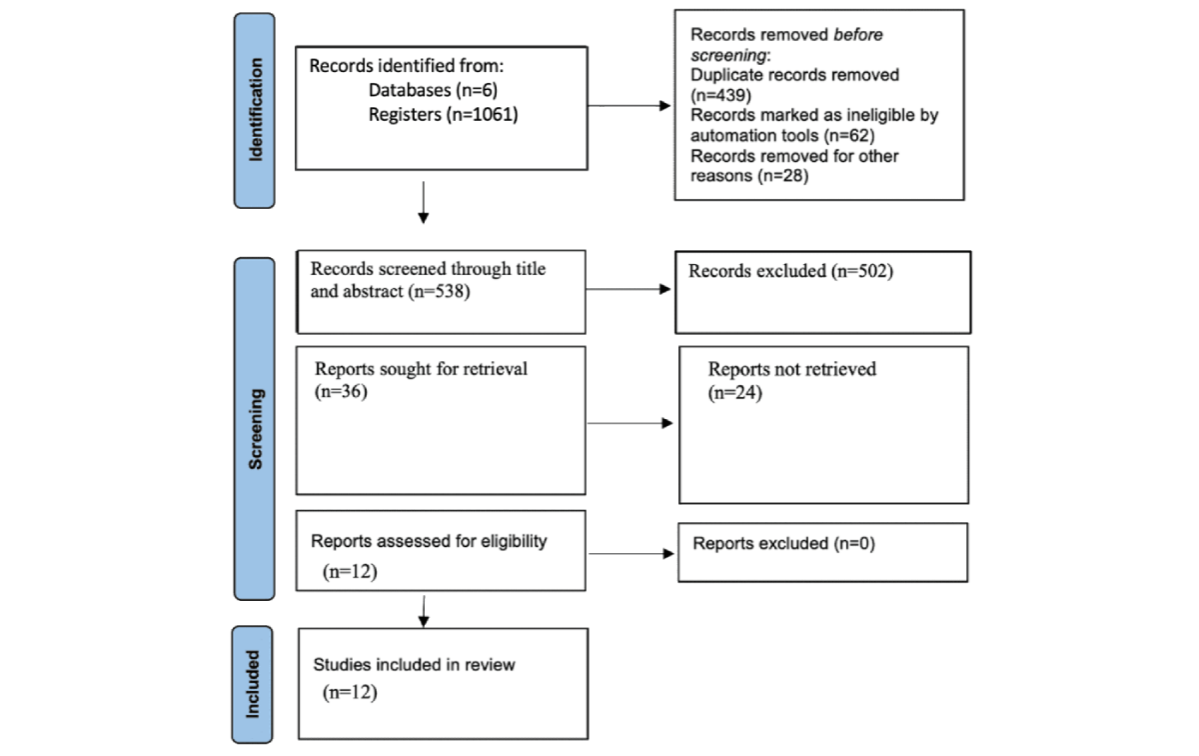
PRISMA (Preferred Reporting Items for Systematic Reviews and Meta Analyses) diagram of studies identified via registers and databases.

After removing duplicates, 538 papers were screened through review of title and abstract. Of these, 36 studies were screened by review of the full text, resulting in 12 papers that met the inclusion criteria: 5 for gestational diabetes mellitus (GDM), 4 for fetal growth, 2 for pulmonary function, and 1 for nervous system disorder. Reasons for exclusion at this stage were recorded and can be found in the flow diagram in Figure 2.

### Quality Assessment

The results of the NHLBI assessment are reported in Figure 1. The research objectives and questions were clear in all the studies. The study population was clearly specified for each study. The participation rate of the eligible individuals and whether the outcome assessors were blinded were not mentioned in any of the studies. Sample size justifications, power descriptions, or variance and effect estimates were not provided or were not clear in 10 of 12 (83%) studies.

### Study Characteristics and Synthesis of the Results

#### Gestational Diabetes Mellitus (GDM)

In all studies, an oral glucose tolerance test (OGTT) was performed for either diagnosis or to confirm diagnosis of GDM. The control group included either analogous non-GDM pregnant women or nonpregnant women. In the majority of the studies (4/5, 80%), the pregnant control and GDM groups were analogous in terms of other health-related factors and sociodemographic characteristics, with GDM as the major difference between the 2 groups. Of the studies, 80% (4/5) suggested dietary and exercise or insulin therapy for the GDM group. All studies measured both frequency and time domains of the HRV assessment. In addition, 80% (4/5) of the studies specified the HRV assessment duration, and it varied between 6 minutes and 8 hours. An electrocardiogram (ECG) was used to assess HRV in 60% (3/5) of the studies. However, the type of spectrum analyzer was either not reported or varied from study to study. The gestational age was reported in 4 of the 5 (80%) studies in which the measurement was performed; the HRV measurement was conducted during pregnancy (all in the third trimester) with additional postpartum (within 3 months of childbirth) assessment in 2 of these studies.

There was no difference between GDM and non-GDM pregnant individuals in terms of HRV metrics in 80% (4/5) of the studies. In the remaining study, frequency domain components varied in terms of changes between control and GDM groups; LF (nu) was higher, and HF (nu) was lower in women with GDM than in pregnant controls [22]. For more information, see Table 1.

**Table 1:** Gestational diabetes mellitus (GDM) and heart rate variability (HRV; n=292).

Author, year, country	Cases	Controls	Study design	Measures	Findings
Weissman et al [23], 2006, Israel	12 pregnant with GDM at GA^a^ of 24-28 weeks; mean age: 31.2 (SD 4.4) years; mean BMI: 26.4 (SD 2.2) kg/m^2^; exclusion criteria: hypertension, thyroid disease, obesity, or a family history of diabetes; no smoking with no suggested diabetic management	15 pregnant without GDM (positive OGCT^b^ and negative OGTT^c^ tests); mean age: 30.8 (SD 3.7) years; mean BMI: 26.7 (SD 2.7) kg/m^2^; analogous age, weight, BMI, and health status as the case group	Cross-sectional: outcome of HRV (VLF^d^, LF^e^, HF^f^, TP^g^, RMSSD^h^, SDNN^i^, SDANN^j^) was assessed for 10 min before the OGTT (phase 0) and was repeated at 60 min after glucose ingestion (phase 1); exposure: OGTT was measured once between 8 AM and 9 AM.	HRV: ECG^k^ with a 12-bit analog-to-digital converter and autoregressive model for IBI^l^; GDM: Hitachi 747 method; OGCT for initial test and confirmation with OGTT (fasting, 1 hour, and 2 hours; at least two >normal)	No statistically significant changes in the TD^m^ measures (TP, RMSSD, SDNN, SDANN) between the 2 groups in the different phases of the study. FD^n^ metrics (LF and HF) in both groups decreased in phase 1; only HF changed significantly (without GDM: mean 104.5, SD 57.4; *P*<.01; with GDM: mean 78.7, SD 62.1; *P*<.01). LF (nu) increased (without GDM: mean 66.8, SD 9.9; with GDM: mean 70.1, SD 17.7; *P*<.05), and HF (nu) decreased (without GDM: mean 33.2, SD 9.9; with GDM: mean 29.9, SD 17.7; *P*<.05) in phase 1 with no significant difference between the 2 groups. LF/HF was higher in the GDM group in phase 0 (mean 3.0, SD 1.9) and increased in both groups in phase 1 (without GDM: mean 2.8, SD 2.1; with GDM: mean 3.9, SD 2.3; *P*<.04), with no statistical difference in changes between groups.
Heiskanen et al [24], 2010, Finland	51 pregnant with GDM; mean age: 31 (SD 1) years; dietary modification for diabetes suggested and insulin received, if necessary	28 pregnant without GDM; mean age: 32 (SD 1) years	Longitudinal: exposure of GDM diagnosed at the beginning of the 3rd trimester; outcomes of glucose, insulin, and HRV (VLF, LF, HF, TP) were measured for 10 min during the 3rd trimester of pregnancy and 3 months postpartum.	GDM: hexokinase method; OGTT (fasting, 1 hour, and 2 hours; at least one >normal) and confirmed with blood glucose profile (every 4 hours per 24 hours); continuous assessment was done (3 days/week); HRV: ECG using fast Fourier transform was used for power spectral estimates of HRV.	TP (pregnancy: mean 1183, SD 180; postpartum: mean 4036, SD 1219; *P*<.01), VLF (pregnancy: mean 417, SD 80; postpartum: mean 516, SD 84; *P*<.05), LF (pregnancy: mean 166, SD 24; postpartum: mean 374, SD 75 *P*<.001), HF (pregnancy: mean 480, SD 113; postpartum: mean 3002, SD 1113; *P*<.01), HF (nu; pregnancy: mean 0.64, SD 0.03; postpartum: mean 0.76, SD 0.03; *P*<.01) were lower during pregnancy than postpartum. LF (nu; pregnancy: mean 0.35, SD 0.03; postpartum: mean 0.22, SD 0.03; *P*<.01) and LF/HF (pregnancy: mean 91, SD 18; postpartum: mean 35, SD 6; *P*<.05) were higher during pregnancy than postpartum. There were no differences between GDM and control groups in HRV components during the third trimester and 3 months postpartum.
Pöyhönen-Alho et al [22], 2010, Finland	41 Caucasian pregnant with GDM (18 with hypertension, 23 without hypertension); mean age: 34.0 (SD 5.6) years; mean BMI: 30.6 (SD 6.0) kg/m^2^; exclusion criteria: smoking and any medication affecting glucose metabolism or the sympathetic nervous system; dietary modification for diabetes suggested and insulin received, if necessary	22 healthy pregnant controls (PC); mean age: 29.5 (SD 4.9) years; mean BMI: 26.9 (SD 3.0) kg/m^2^; and 14 healthy nonpregnant controls (NPC); mean age: 30.4 (SD 6.9) years; mean BMI: 26.5 (SD 6.3) kg/m^2^; analogous BMI and exclusion criteria as the case group	Cross-sectional: exposures of GDM diagnosed in pregnancy; outcome of HRV (SDNN, SDANN, LF, HF, VLF) was assessed once at night (11 PM through 8 AM for 8 hours)	GDM: OGTT (fasting, 1 hour, and 2 hour); HRV: Holter	No difference between 2 GDM and PC groups except at 4 AM: LF (nu) was higher (GDM: mean 60.0, SD 12.4; PC: mean 48.5, SD 12.8; *P*=.01), and HF (nu; GDM: mean 40.0, SD 12.8; PC: mean 51.5, SD 12.8; *P*=.01) and HF power (GDM: mean 842.7, SD 652.6; PC: mean 1053.4, SD 1024.4; *P*=.02) were lower. There were differences between GDM and NPC for the following: SDNN (GDM: mean 87.6, SD 23.8; NPC: mean 136.1, SD 26.9; *P*<.001), SDANN (GDM: mean 62.6, SD 18.9; NPC: mean 103.8, SD 23.7; *P*<.001), LF (GDM: mean 1178.5, SD 655.1; NPC: mean 3062.9, SD 1789.0; *P*<.001), HF (GDM: mean 842.7, SD 652.6; NPC: mean 1631.4, SD 1201.4; *P*=.02), and VLF (GDM: mean 3259, SD 1646; NPC: mean 5897, SD 1614; *P*<.001). In all 3 groups, changes in LF (nu), HF (nu), and LF/HF remained unchanged during the 8-hour time frame.
Maser et al [25], 2014, United States	31 pregnant with GDM; mean age: 32 (SD 4) years; mean BMI**:** 35.2 (SD 7.9) kg/m^2^; exclusion criteria: type 1 or 2 diabetes mellitus, pregestational hypertension, preeclampsia during current pregnancy, preterm labor, cervical shortening, current multiple fetuses, cardiopulmonary diseases; dietary and exercise modification for diabetes suggested and insulin received, if needed	12 without GDM; mean age: 30 (SD 5) years; mean BMI: 30.9 (SD 6.4) kg/m^2^; analogous with case group; exclusion criteria: the same as the case group in terms of age, blood pressure, BMI, and GA	Longitudinal: exposures of GDM diagnosed at GA of 30-35 weeks; outcome of overnight HRV (LF, HF, TP) was assessed for 6 min during the mid-third trimester (GA: 30-35 week) and 2-3 months postpartum	HRV: ANX 3.0 (ANSAR Medical Technologies Inc); GDM: OGCT for initial test and confirmation with OGTT (fasting, 1 hour, and 2 hours; at least two >normal); continuous glucose assessment was performed once a week.	There was no difference between GDM and control groups during late pregnancy and after delivery for any HRV metrics including normalized and nonnormalized metrics.
Žákovičová et al [26], 2014, Czech Republic	35 pregnant with GDM; mean age: 32 (SD 4) years; mean BMI: 28.2 (SD 3.8) kg/m^2^; exclusion criteria: history of hypertension, preeclampsia, and chronic diseases except for controlled hypothyroidism; dietary modification for diabetes suggested and insulin received, if necessary	31 pregnant without GDM; mean age: 30.3 (4.2 years); mean BMI: 27.1 (SD 4.1) kg/m^2^; analogous in weight, height, BMI, and age with the case group	Cross-sectional: exposures of GDM diagnosed in the GA of 24-28 weeks; outcome of HRV (LF, HF, TP) was measured at GA of 36 weeks	GDM: OGCT was used to test for GDM; cases were confirmed with OGTT (fasting, 1 hour, and 2 hours; at least 1 >normal); OGTT was continued biweekly until childbirth; for HRV, ECG was used, and the VariaCardio TF4 device and Fourier transform algorithm were used for analysis.	TP, HF, and LF/HF did not differ between the 2 groups.

^a^GA: gestational age.

^b^OGCT: oral glucose challenge test.

^c^OGTT: oral glucose tolerance test.

^d^VLF: very low frequency.

^e^LF: low frequency.

^f^HF: high frequency.

^g^TP: total power.

^h^RMSSD: root mean square of successive differences between normal heartbeats.

^i^SDNN: standard deviation of the normal-to-normal R-R intervals.

^j^SDANN: standard deviation of the average normal-to-normal (NN) intervals.

^k^ECG: electrocardiogram.

^l^IBI: interbeat interval.

^m^TD: time domain.

^n^FD: frequency domain.

#### Fetal Growth

The study populations were mainly African (American or non-American), non-Hispanic White, Latino, and German. In 75% (3/4) of the studies, the population was healthy excluding the potential risk factors for ANS; of these, 1 study, however, included obese or overweight women as more than 50% of its population. In 1 of the 4 studies (25%), the population had hypertensive disorder along with fetal growth retardation. The considered HRV metrics, including the time domain (SDNN, RMSSD) and frequency domain (LF, HF, VLF), varied from study to study. HRV assessment was conducted in the second or third trimester; the assessment frequency varied from 1 time to 5 times among the studies, with 50% (2/4) of the studies conducting an assessment 1 time. The assessment duration varied from 10 minutes to 55 minutes in the studies. Fetal growth was assessed based on birth weight or *z* score for birth weight in 75% (3/4) of the studies. One study, however, used small for gestational age as the measurement of fetal growth. ECG was used for HRV assessment in all the studies, but the applied standards varied from study to study.

In the studies, among the considered HRV metrics, SDNN, RMSSD, and HF had a significant negative association with fetal growth. In 75% (3/4) of the studies, RMSSD, as the commonly assessed metric, had a significant negative association with fetal growth. In 1 of the studies that included White and African American women as the 2 study populations, HRV metrics did not differ with fetal growth in the African American group [27]. Also, in 1 of the studies [28], 2 case groups were included, both with impaired uterine perfusion; 1 group included poor outcome (eg, hypertensive disorder, impaired fetal growth), and the other included no poor outcomes. In the case group with poor outcomes, failing to perform a separate analysis on each poor outcome in terms of its independent associations with HRV metrics hindered the understanding of the actual association between fetal growth and autonomic function. This is problematic, as hypertensive disorder is known to be linked to autonomic dysfunction, which could mask the impact of fetal growth on ANS function. Thus, a pooled analysis without distinguishing the outcomes can threaten the specificity and therefore the validity of the findings. For more information, see Table 2.

**Table 2:** Fetal growth and heart rate variability (HRV; n=5988).

Author, year, country	Case	Control	Study design	Measures	Findings
Odendaal et al [29], 2019, South Africa	5655 pregnant in the first trimester (GA^a^: ≥6 weeks) between 16 years and 45 years old, singleton; prior history of heart disease, hyperthyroidism, diabetes, and placental abruption was found in <1%; hypertension was found in 12.1%; preeclampsia was found in 3.9%; mean age 24.5 (SD 6.0) years; mean BMI 25.5 (SD 5.7) kg/m^2^; exclusion criteria: N/A^b^	N/A	Retrospective study using data from 2007-2015; exposure of HRV (SDNN, RMSSD) was assessed for 36-55 minutes at 3 times, at 20-24, 28-32, and ≥34 weeks; outcome of birth weight was assessed on a case report form.	HRV: ECG^c^ with 5 electrodes was used and imported into MATLAB. Dawes-Redman guidelines were used to quantify IBI^d^ features. Artifact management and sparsity-based epoch rejection were used for data preprocessing. Fetal growth: birth weight and *z* score	At both 20-24 weeks and ≥34 weeks, birth weight correlated positively with maternal heart rate but negatively with SDNN^e^ (Spearman correlation=−0.0437; *P*<.02) and RMSSD^f^ (Spearman correlation=−0.0627; *P*<.01).
Christian et al [27], 2021, United States	39 pregnant (19 African American; 20 White), at GA of 21-24 weeks; exclusion criteria: tobacco/drug use; chronic diseases such as endocrine, cardiovascular, cancer, diabetes, hypertensive disorder, anemia, medication use (psychotic, antibiotic), and excessive caffeine use; BMI ≥30 kg/m^2^	N/A	Retrospective study; secondary data analysis between 2009 and 2011; exposure of HRV (HF^g^) was assessed for 10 min in the afternoon once in the second trimester; outcome of birth weight was assessed once right after childbirth using the medical record.	HRV: ECG (Task Force of the European Society of Cardiology) was used for signal capturing and artifact management; offline signal inspection with Mindware Technology’s HRV 2.51 software; fetal growth: birth weight (grams) collected postdelivery and from the medical chart	White group had a negative relationship between HF and birth weight (correlation coefficient=–0.757, *P*=.002); no relationship was found in the African American group.
Ecklund-Flores et al [30], 2016, United States	227 pregnant; GA: 36 weeks; 50% obese or overweight; 54% primigravidae, singleton; 68% Latino; mean age 26.45 (SD 6.02) years; mean BMI 25.32 (SD 4.99) kg/m^2^; exclusion criteria: no GDM, hypertension, or other related medical conditions, and no cigarette, alcohol, or drug use during pregnancy	N/A	Longitudinal observational study: outcome of birth weight adjusted for GA at birth and sex; exposure of adjusted HRV (RMSSD) was assessed for 5 min at baseline (GA: 36 weeks)	HRV: ECG digitized at 500 Hz (National Instruments 16XE50); fetal growth: birth weight was assessed after birth, and the data collection method was not reported.	RMSSD and birth weight had significant negative associations (Pearson correlation: –0.13; *P*<.001).
Voss et al [28], 2006, Germany	16 pregnant with abnormal uterine perfusion and normal outcome (AP-NO), age: 29 (range 28 to 33) years; 19 women with abnormal uterine perfusion and pathologic outcome (AP-PO; small for GA, gestational hypertension, preeclampsia), singleton, age: 26 (range 25 to 30) years	32 healthy pregnant with normal uterine perfusion (CON), age: 28 (24 to 31) years; analogous to the case group in maternal age, gravidity, and parity	Longitudinal observational study: exposure of HRV (normalized LF^h^, VLF^i^, mean NN^j^, SDNN, RMSSD) for 30 min between 8 AM and 12 PM, 5 times during pregnancy (GA: 18-22, 23-26, 27-30, 31-34, 35-37weeks); outcome of small for GA was assessed (birth weight <10th percentile of an own-reference group).	Fetal growth: data collection record was not reported; HRV: ECG (1600 Hz) based on task force standards	RMSSD decreased (*P*=.009), and VLF (*P*<.001) and LF (n; *P*=.003) increased in CON during pregnancy. No HRV parameter changed significantly in the course of gestation in AP-NO and AP-PO. No intergroup differences in HRV were found between CON and AP-NO. The comparison of AP-PO with CON and AP-NO, however, revealed a significant increase in mean NN (*P*=.03) as well as RMSSD (CON vs AP-PO: *P*=.008; AP-NO vs AP-PO: *P*=.01). AP-PO group had a significantly increased SDNN compared with AP-NO (*P*=.03). Effect measure amount was not clear.

^a^GA: gestational age.

^b^N/A: not applicable.

^c^ECG: electrocardiogram.

^d^IBI: interbeat interval.

^e^SDNN: standard deviation of the normal-to-normal R-R intervals.

^f^RMSSD: root mean square of successive differences between normal heartbeats.

^g^HF: high frequency.

^h^LF: low frequency.

^i^VLF: very low frequency.

^j^NN: N-N interval.

#### Cardiovascular and Hemodynamic Variables

To avoid redundancy and provide a comprehensive overview of the literature, we incorporated the findings of a previously published systematic review that explored the association between hypertensive disorders and HRV. We did not include the review among our reviewed studies in the Results section but included a summary of the review's main results and conclusions in the Discussion section of our review and Multimedia Appendix 3 to provide transparency and facilitate replication.

#### Other

Other results were related to the pulmonary system and the central nervous system.

Regarding the pulmonary system, Amador-Licona et al [31] conducted a study to investigate cardiovascular autonomic and pulmonary function in obese pregnant women; 178 pregnant women with no chronic diseases were included. The study measures were assessed using spirometry, 10-minute oximetry, and 60-minute ECG monitoring twice during pregnancy. All assessments were performed between 8 AM and 10 AM to maintain consistency in the measurements. Their findings indicated that, in the obese group, the change in forced expiratory volume at 1 second to forced vital capacity (FEV1/FVC) during pregnancy was linked to the LF/HF metric in the third trimester after adjusting for confounding factors such as insulin, weight gain, and blood pressure (Table 3).

Liao and Jaw [32] also studied the potential of HRV analysis to assess progress in amniotic fluid embolism and disseminated intravascular coagulopathy. They compared 2 cases with 105 healthy pregnant women. Entropy (a nonlinear HRV metric) was used to assess HRV and significantly decreased in amniotic fluid embolism and disseminated intravascular coagulopathy and increased in the intensive care unit during recovery. They concluded that entropy analysis has the potential to be applied to monitor amniotic fluid embolism and the progress of disseminated intravascular coagulopathy in a patient (Table 3).

Regarding the central nervous system, a case was reported in Germany, suggesting the ANS is dysregulated right before and during the grand mal seizure [33]. The case was a 21-year-old gravida 1 para 0 who had epilepsy due to a frontotemporal arteriovenous malformation and was on anticonvulsant medication. The HRV was assessed twice, and the results indicated no changes in week 20; however, during the second monitoring session (week 24) when the patient developed a grand mal seizure, HRV was significantly altered. At the beginning of the monitoring (12 minutes prior to seizure), VLF increased, LF was delayed, and HF remained unchanged. HF, however, started to increase afterward.

**Table 3:** Heart rate variability (HRV) and other complications such as pulmonary disease or seizures (n=376).

Author, year, country			Case		Control		Study design		Measures			Findings
**Pulmonary system (n=375)**												
	Amador-Licona et al [31], 2009, Mexico	178 pregnant women (88 obese, BMI>27 kg/m^2^); age: 28.2 years; exclusion criteria: no chronic diseases (eg, hypertension, diabetes)		90 nonobese (BMI >18.5 kg/m^2^ and <24.9 kg/m^2^); age: 26.6 years; exclusion criteria: no chronic diseases (eg, hypertension, diabetes)		Longitudinal: exposures of spirometry, 10-min oximetry; outcome of HRV by 60-minute electrocardiograph monitoring; both exposure and outcome were assessed twice between 8 AM and 10 AM during pregnancy, at GA^a^ of 24-28 weeks and 36-37 weeks		Spirometry (using EasyOne 2001-2 spirometer, NDD) and oximetry (using the Onix 2001 oximeter; HRV using a 3-channel Holter recorder (model GBI-3S, Galix Biomedical Instrumentation Inc)				Change in FEV1/FVC during pregnancy was linked to the LF^c^/HF^d^ metric in the third trimester after adjusting for confounding factors such as insulin, weight gain, and blood pressure (β=−0.42; *P*<.001).
	Liao and Jaw [32], 2011, Taiwan	n=2; case 1 (sudden acute dyspnea with cyanosis): 36-year-old primigravid woman was admitted in active labor with no history of epilepsy, cardiopulmonary, or renal disease; caes 2 (acute dyspnea with cyanosis): 35-year-old, gravid 2, para 1 woman was admitted at 37 weeks of gestation due to labor pain and ruptured membranes.		105 healthy pregnant women with no complication during labor		Longitudinal: exposures of amniotic fluid embolism and disseminated intravascular coagulopathy; outcome of HRV (entropy) was assessed continuously after admission to labor until recovery form the complication.		Amniotic fluid embolism and disseminated intravascular coagulopathy: specialist’s diagnosis; HRV: ECG^e^				HRV significantly decreased in amniotic fluid embolism and disseminated intravascular coagulopathy and increased in ICU^f^ during recovery from these complications.
**Central nervous system (n=1)**												
	Faber et al [33], 2004, Netherland	n=1; 21-year-old gravid 1, para 0 woman had epilepsy due to a frontotemporal arteriovenous malformation in the 24th week of gestation		N/A^g^		Longitudinal: exposure of HRV (frequency domain: VLF^h^, LF, HF, LF/HF) was assessed starting from 20 weeks of GA through 24 weeks of GA for 30 minutes every 4 weeks and for 12 minutes until the onset of seizure; outcome of grand mal seizure				HRV: N/A; grand mal seizure: tonic-clonic convulsions diagnosed by a specialist	HRV showed significant alterations at 12 minutes prior to the seizure: VLF increased; LF was delayed, and HF remained unchanged. HF, however, started to increase afterward.	

^a^GA: gestational age.

^b^FEV1/FVC: forced expiratory volume at 1 second to forced vital capacity.

^c^LF: low frequency.

^d^HF: high frequency.

^e^ECG: electrocardiogram.

^f^ICU: intensive care unit.

^g^N/A: not applicable.

^h^VLF: very low frequency.

#### Difference in HRV Changes in Pregnancy Complications

The significance of changes in various HRV components varied from complication to complication. However, impaired fetal growth and hypertensive disorder reached common significance in an HRV component (RMSSD) based on the majority of the studies but in the opposite direction. This reverse association supports studies that linked a high birth weight to maternal hypertension [34-37]. See more information in Table 4.

**Table 4:** Heart rate variability (HRV) components in pregnancy complications, with the difference in HRV based on the majority of studies.

Pregnancy complications			Linear HRV component in the frequency domain (FD)															Linear HRV component in the time domain (TD)									Nonlinear HRV component
			HF^a^		LF^b^ (nu)		LF		HF (nu)		LF/HF		LF/HF (ln)		VLF^c^		TP^d^			SDNN^e^		RMSSD^f^		SDANN (ln)		Entropy	
GDM^g^ (4 of 5 studies)			Not significant^h^		Not significant^h^		Not significant^h^		Not significant^h^		Not significant^h^		Not significant^h^		Not significant^h^		Not significant^h^			Not significant^h^		Not significant^h^		Not significant^h^		Not significant^h^	
Impaired fetal growth (4 of 4 studies)			—^i^		—		—		—		—		—		—		—			—		Increased		—		—	
**Hypertensive disorders (n=1) that were reviewed in 24 studies**																											
	GH^j^ (n≥12)	Decreased		Increased		—		—		—		Increased		—		Decreased			Decreased		Decreased		—		—		
	Preeclampsia (n≥12)	Decreased		Increased		—		Decreased		Increased		Increased		—		—			Decreased		—		Increased		—		
	HPD^k^ (n≥12)	Not significant^h^		Not significant^h^		Not significant^h^		Not significant^h^		Not significant^h^		Not significant^h^		Not significant^h^		Not significant^h^			Not significant^h^		Not significant^h^		Not significant^h^		Not significant^h^		
**Pulmonary function (n=2)**																											
	Low FEV1/FVC^l^ (n=1)	—		—		—		—		Increased		—		—		—			—		—		—		—		
	Amniotic fluid embolism (n=1)	—		—		—		—		—		—		—		—			—		—		—		Decreased		
**Central nervous system (n=1)**																											
	Grand mal seizure	—		—		Decreased		—		—		—		Increased		—			—		—		—		—		

^a^HF: high frequency.

^b^LF: low frequency.

^c^VLF: very low frequency.

^d^TP: total power.

^e^SDNN: standard deviation of the normal-to-normal R-R intervals.

^f^RMSSD: root mean square of successive differences between normal heartbeats.

^g^GDM: gestational diabetes mellitus.

^h^The common biomarkers in the majority of the studies showed no significant difference from the controls.

^i^Not assessed by the majority of the studies.

^j^GH: gestational hypertension

^k^HPD: hypertensive disorders

^l^FEV1/FVC: forced expiratory volume at 1 second to forced vital capacity.

## Discussion

### Principal Findings and Comparison With Existing Literature

The findings of this study support potential ANS dysregulation in some pregnancy complications including hypertensive disorder and fetal growth.

#### Cardiovascular and Hemodynamic

These adaptations in the cardiovascular system include adjusting blood pressure, blood volume, and heart activity. Systemic vasodilation is the primary initial hemodynamic event that begins during the luteal phase of the menstrual cycle following the release of chemical mediators by the corpus luteum [9-11]. These hemodynamic adaptations provide the required blood for fetal needs of growth [13] through increased sympathetic tone and parasympathetic withdrawal.

Moors et al [38] conducted a systematic review on the difference in autonomic function using HRV between hypertensive and normotensive pregnancies. Higher LF/HF and lower HF and RMSSD were found in the hypertensive group, compared with the normotensive pregnant controls. This can be explained by overactivation of sympathetic over parasympathetic tone resulting from functional or structural damage of the vascular or nervous system. As we discussed earlier, ANS physiologically regulates hemodynamics (eg, blood volume, cardiac output) in response to general vasodilation and consecutive hypotension. This requires sympathetic activation and, thus, sympathetic dominance and parasympathetic withdrawal. Failure of the ANS to function in response to decreased blood pressure following pregnancy-induced systemic vasodilation may increase the risk of hypertensive disorders.

#### GDM

Pregnancy affects both ANS function and metabolic regulations [1,39,40]. In addition to ANS alterations during pregnancy, a diabetogenic effect on metabolism has been indicated in pregnant women. Placental-derived hormones induce this impact by reprogramming maternal physiology to achieve an insulin-resistant state, by reducing insulin sensitivity [41]. This, in turn, increases the risk of developing new diabetes (GDM) or worsening existing diabetes during pregnancy.

Due to the destructive impact of diabetes on the ANS, intensified diabetic neuropathy in pregnant women with GDM is expected compared with non-GDM pregnant and nonpregnant individuals. In this review study, however, an association between GDM and maternal ANS during pregnancy and postpartum was not supported. Part of this can be due to the short assessment duration. According to the literature about HRV assessment protocols, any duration less than 24 hours (circadian rhythm) lacks the ability to capture reliable HRV metrics and, thus, ANS function [15]. Longer recording epochs better represent slower fluctuations affected by circadian rhythms as well as the cardiovascular response to a wider range of environmental stimuli. Interestingly, in this review, studies that assessed HRV for a shorter period (minutes) showed no difference between pregnant women with GDM and without GDM, whereas those with a longer assessment time (hours) indicated a difference between the 2 groups. For example, Pöyhönen-Alho et al [22] assessed HRV for 8 hours and indicated significant differences between the 2 groups in terms of frequency domain metrics.

Additionally, most of the studies often assessed GDM and autonomic function in a cross-sectional or longitudinal method with a short interval between the 2 assessments (GDM diagnosis and HRV assessment), failing to consider a period for the autonomic system to be affected. This consideration is important, as GDM can be asymptomatic for the long term and may not indicate a significant influence on the ANS in the short term. One of the potential solutions may be the use of glycated hemoglobin to represent glycemic control in the long term. The impact of long-term diabetes control on HRV is more valuable and clinically useful to study than diagnosed diabetes, as it focuses on control rather than treatment for diabetes, which is an often chronic and noncurable condition and relies on symptom management. In the reviewed studies, 80% suggested dietary modification for diabetes, insulin therapy, or exercise for people with diabetes. This manipulation may have impacted the findings of the studies, resulting in no difference in ANS function in people with diabetes. This manipulation leads to a failure in representing actual glycemic control following the autonomy of the population in real life.

#### Fetal Growth

Our study indicates that there is a negative relationship between fetal development and vagal tone during pregnancy. This association can be partially explained by the fetal-maternal physiology for developmental adaptations during pregnancy. The uterine complex, including the placenta, is a multifunctional organ, representing the vital interface between the mother and fetus, and placental blood circulation holds the link between the mother and fetus. The required supply of oxygen and nutrients for fetal development is maintained via uteroplacental perfusion. On the other hand, circulation and transportation of the supply via blood flow are regulated by the ANS by adjusting vascular resistance. Sympathetic and parasympathetic tone determine the vascular tone in response to hemodynamic dysregulations following pregnancy-induced demands. Any changes in this tone can affect uterine perfusion by increasing or decreasing uteroplacental vascular resistance. Impaired perfusion, if it lasts long or occurs frequently, in turn can cause less supply transfer and, thus, lower birth weight [42,43]. It is worth mentioning that this uteroplacental autonomic regulation is originally for fetal protection against harmful maternal products (eg, cortisol and insulin). This protective mechanism, however, can result in poor fetal outcomes by reducing the essential blood supply to the fetus. Our findings, however, challenge studies that have linked higher sympathetic tone to low birth weight. Our review indicates that increased vagal tone is associated with low birth weight, which is in conflict with the theory that suggests the potential of increased sympathetic tone for explaining low birth weight by disturbing uteroplacental perfusion.

Interestingly, maternal autonomic and fetal autonomic tone have also been linked in a recent study [44]. This association can be explained by vascular resistance of the uteroplacental pathway following maternal ANS regulation in response to fetal developmental demands. This mechanism may lead to fetal autonomic regulation in response to the changed perfusion. This finding, if supported by more relevant literature, may indicate the potential of maternal ANS function to predict fetal autonomic regulation.

#### Complication Co-occurrence

It is evident that pregnancy complications are often correlated and tend to co-occur. For example, individuals with GDM may increase the chance of developing hypertensive disorder by 30% [45-48], which is explainable by diabetic neuropathy and vascular damage. Indeed, vascular impairment resulting from neuropathy may lead to hemodynamic dysregulation and, thus, hypertensive disorder. These two conditions, GDM and hypertensive disorders, can also impact other pregnancy complications such as fetal growth. For example, 15% to 45% of babies born to mothers with diabetes have macrosomia [47], which also occurs in babies born to hypertensive pregnant women [34-37]. This correlation explains how fetal development can be affected by both GDM and hypertensive disorders. To explain the uncertainty in the responsiveness of the ANS to GDM in this review, the stage of diabetic neuropathy may be the determinative factor. To understand this critical stage when the hemodynamic dysregulation starts to occur, a continuous ANS assessment is required to indicate when and how GDM may lead to neuropathy and vascular damage and thus ANS tone alteration.

### Strengths and Limitations

This study is a PRISMA-guided systematic review that sheds light on the utilization of HRV in representing ANS dysregulation and its possible link with pregnancy complications. Although this study provides insight in understanding the potential pathway for pregnancy complications, a meta-analysis was not conducted due to the divergent time periods used for the measurement. Another limitation of this study is that it was not registered prior to beginning the review.

In terms of using HRV, HRV has been suggested as a health index for various concepts such as mental distress, physical activity, and meditation. Although these suggestions can be accepted, considering the aforementioned impact on ANS regulation, it may affect the specificity of HRV for reflecting the ANS. Careful reconsideration is needed to define biomarkers for these concepts. Additionally, in the evidence, there is a concern that HRV may only reflect the cardiac vagal tone and not necessarily sympathetic activity. Although most of the HRV metrics represent parasympathetic activity, there are still metrics that reflect the sympathetic/parasympathetic balance. In addition, due to the dynamic balance between sympathetic and parasympathetic activity, it is expected that the activity of each branch can predict the other, acting like the 2 sides of a homeostasis seesaw.

One of the advantages of using HRV to assess the ANS is the ability to continuously assess using wearable smart technologies. This continuity in the assessment may increase the reliability of the assessments in reflecting pregnancy and the ANS. This is due to the dynamic and ever-changing nature of both pregnancy and the ANS that cannot be represented by episodic and short-term assessments. HRV also can be assessed cost-effectively and noninvasively, reflecting the real-life function of the ANS.

### Conclusion and Implications

Due to the divergent HRV bands considered for assessment in the different studies, it was not practical to compare the studies comprehensively. However, our findings, which are based on the majority of studies for each complication (within-group), suggest that ANS function has been associated with some common pregnancy complications including hypertensive disorder and fetal growth. However, existing studies do not support an association between the ANS and GDM. Studies that have linked pulmonary and central nervous system disorders to ANS function did not provide sufficient evidence to draw conclusions. More studies are needed to understand how the ANS, through HRV, is associated with other systems during pregnancy. Future studies are suggested to cover the methodological gaps in HRV assessment (eg, short assessment duration, noncontinuous assessment, low-quality standards) to represent more reliable findings.

## Appendix

Multimedia Appendix 1 Heart rate variability (HRV) components and metrics.

Multimedia Appendix 2 Search strategy.

Multimedia Appendix 3 Hypertensive disorders and heart rate variability.

## Abbreviations

ANS: autonomic nervous system
ECG: electrocardiogram
FEV1/FVC: forced expiratory volume at 1 second to forced vital capacity
GDM: gestational diabetes mellitus
HF: high frequency
HRV: heart rate variability
LF: low frequency
NHLBI: National Heart, Lung, and Blood Institute
PECO: Population, Exposure, Comparison or Controls, and Outcome
PNS: parasympathetic nervous system
PRISMA: Preferred Reporting Items for Systematic Reviews and Meta-Analyses
RMSSD: root mean square of successive differences between normal heartbeats
SDNN: standard deviation of the normal-to-normal R-R intervals
SNS: sympathetic nervous system
VLF: very low frequency
