# Supplementary material for: Heart Rate Variability and Pregnancy Complications: Systematic Review
Source: Interact J Med Res. 2023 Jun 5;12:e44430. doi: 10.2196/44430 (PMC10280337; doi:10.2196/44430)
Supplement: Multimedia Appendix 1 [file ijmr_v12i1e44430_app1.docx]

**Multimedia Appendix 1**

**Table S1.** HRV components and metrics.

| **Components** | **Metrics** | **Unit** | **Description** |
| --- | --- | --- | --- |
| **Time domain** | SDNN | ms | Standard deviation of NN interval |
|  | RMSSD | ms | Root mean square of successive NN interval differences |
|  | NN50 | ms | Mean number of times an hour in which the change in successive normal sinus (NN) intervals exceeds 50 ms |
|  | pNN50 | % | Percentage of successive NN interval that differ by more than 50 ms |
|  | SDSD | ms | Standard deviation of the differences between successive NN intervals |
|  | HTI | - | Integral of intensity of NN interval histogram divided by its height |
| **Frequency domain** | LF | ms^2^/nu | Absolute/relative power of low frequency band (0.04-0.15 Hz) |
|  | HF | ms^2^/nu | Absolute/relative power of high frequency band (0.15-0.4 Hz) |
|  | LF/HF | % | Ratio of LF to HF |
|  | ULF | ms^2^ | Absolute power of ultra-low frequency band (≤ 0.003 Hz) |
|  | VLF | ms^2^ | Absolute power of very-low frequency band (0.0033-0.04Hz) |
|  | Avg NN | ms | Mean of NN intervals |
|  | TP | ms^2^ | Absolute power of total frequency band (≤0.4 Hz) |
| **Nonlinear** | SD_1_ | ms^2^ | Poincare plot standard deviation perpendicular the line of identity |
|  | SD_2_ | ms^2^ | Poincare plot standard deviation along the line of identity |
