# Supplementary material for: Heart Rate Variability and Pregnancy Complications: Systematic Review
Source: Interact J Med Res. 2023 Jun 5;12:e44430. doi: 10.2196/44430 (PMC10280337; doi:10.2196/44430)
Supplement: Multimedia Appendix 2 [file ijmr_v12i1e44430_app2.docx]

**Multimedia Appendix 2**

**Table S2.** Search strategy.

| **Database** | **Search strategy** | **Search time span** | **Filters** | **Articles#** |
| --- | --- | --- | --- | --- |
| **PubMed 20210805** | ("heart rate variability" OR HRV[tiab]) AND ("Pregnancy Complications"[Mesh] OR "Pregnancy Complication*" OR "Adverse Birth Outcome*" OR "Pregnancy/complications"[Mesh] OR "Pregnancy/pathology"[Mesh] OR "Pregnancy/physiopathology"[Mesh] OR "Pregnancy Outcome/complications"[Mesh] OR "Pregnancy Outcome/pathology"[Mesh] OR "Pregnancy Outcome/physiopathology"[Mesh]) | 1996- Feb 2022 | English language, proceeding papers, book chapter, editorials, conference papers, dissertations, letters | 344 |
| **Medline Complete 20210813 (corrected)** | ("heart rate variability" OR TI HRV OR AB HRV) AND ((MH "Pregnancy Complications+") OR (pregnan* N3 complicat*) OR "Adverse Birth Outcome*" OR (MH "Pregnancy+/CO/PP/PA") OR (MH "Pregnancy Outcome+/CO/PP/PA")) | 1971- Feb 2022 |  | 352 |
| **CINAHL Complete 20210813 (corrected)** | ((MH "Heart Rate Variability") OR "heart rate variability" OR TI HRV OR AB HRV) AND ((MH "Pregnancy Complications+") OR (pregnan* N3 complicat*) OR "Adverse Birth Outcome*" OR (MH "Pregnancy+/CO/PP/PA") OR (MH "Pregnancy Outcomes")) | 1961-Feb 2022 |  | 121 |
| **Web of Science Core Collection 20210813** | (TS="heart rate variability" OR TI=HRV OR AB=HRV) AND TS=(pregnan* NEAR/3 complicat* OR "adverse birth outcome*" OR pregnanc* NEAR/3 outcome*) | 1900-Feb 2022 |  | 57 |
| **Scopus 20210813** | (TITLE-ABS-KEY ("heart rate variability" OR hrv) AND TITLE-ABS-KEY (pregnan* W/3 complicat* OR "adverse birth outcome*" OR pregnanc* W/3 outcome* ) ) ) | 1970-Feb 2022 |  | 141 |
| **Cochrane Library 20220204** | ("heart rate variability" OR hrv:ti  AND  [mh "Pregnancy Complications"] OR [mh "Pregnancy"/CO,PP,PA] OR [mh "Pregnancy Outcome"/CO,PP,PA] OR pregnan* NEAR/3 complicat* OR "adverse birth outcome*" OR pregnanc* NEAR/3 outcome* | 1993-Feb 2022 |  | 46 |
