# Supplementary material for: Heart Rate Variability and Pregnancy Complications: Systematic Review
Source: Interact J Med Res. 2023 Jun 5;12:e44430. doi: 10.2196/44430 (PMC10280337; doi:10.2196/44430)
Supplement: Multimedia Appendix 3 [file ijmr_v12i1e44430_app3.docx]

**Multimedia Appendix 3**

**Table S3.** Hypertensive disorders and heart rate variability (HRV; n=24).

| **HRV Metrics** | **GH vs NT:** | **Pre-eclampsia vs NT:** | **HPD vs NT:** |
| --- | --- | --- | --- |
| TP (normalized [nu], logarithmic [ln]) | TP: No difference in 67% (2 out of 3) studies, and ↓ in 33% (1 out of 3) studies  TP (ln): ↓ in 100% (1 out of 1) study | TP: No difference in 75% (3 out of 4) studies, and ↓ in 25% (1 out of 4) studies  TP (ln): No difference in 50% (1 out of 2), or a ↓ in 50% (1 out of 2) studies | No significant difference was found |
| LF (normalized [nu], logarithmic [ln]) | LF: No difference in 83% (5 out of 6) studies, and ↑ in 16% (1 out of 6) studies  LF (nu): ↑ in 100% (2 out of 2) studies  LF (In): No difference in 50% (1 out of 2) studies, and ↑ in 50% (1 out of 2) studies | LF: No difference in 50% (5 out of 10) studies, ↑ in 20% (2 out of 10) studies, and ↓ in 30% (3 out of 10) studies  LF (nu): ↑ in 75% (3 out of 4) studies, and no significant difference in 25% (1 out of 4) studies  LF (ln): ↑ in 40% (2 out of 5) studies, or no difference in 60% (3 out of 5) studies | No significant difference was found. |
| HF (normalized [nu], logarithmic [ln]) | HF: No difference in 67% (2 out of 3) studies, and ↑ in 33% (1 out of 3) studies  HF (nu): ↓ in 67% (2 out of 3) studies in 1st trimester and remained unchanged in the 2nd and 3th trimesters; HF (nu) ↓ in 33% (1 out of 3) studies at 3th trimester  HF (In): No difference in 100% (2 out of 2) studies | HF: ↑ in 10% (1 out of 10) studies, ↓ in 50% (5 out of 10) studies, and no difference in 40% (4 out of 10) studies  HF (nu): ↓ in 100% (3 out of 3) studies  HF (ln): ↓ in 20% (1 out of 5), or remained unchanged in 80% (4 out of 5) studies | ↑ in 50% (1 out of 2) studies, or no difference in 50% (1 out of 2) studies |
| LF/HF (normalized [nu], logarithmic [ln]) | LF/HF: No difference in 60% (3 out of 5), and ↑ in 40% (2 out of 3) studies  LF/HF (ln): ↑ in 100% (1 out of 1) study | LF/HF: ↑ in 67% (4 out of 6) studies, and no difference in 33% (2 out of 6) studies  LF/HF (ln): ↑ in 75% (3 out of 4) studies, or no difference in 25% (1 out of 4) studies | No difference in 100% (2 out of 2) studies |
| SDNN (normalized [nu], logarithmic [ln]) | SDNN: No difference in 33% (1 out of 3) studies, and ↓ in 67% (2 out of 3) studies  SDNN (ln): ↓ in 100% (1 out of 1) study | SDNN: ↓ in 60% (3 out of 5) studies, and no difference in 40% (2 out of 5) studies  SDNN (ln): ↓ in 50% (1 out of 2) studies, or remained unchanged in 50% (1 out of 2) studies | N.A. |
| SDANN (normalized [nu], logarithmic [ln]) | SDANN (In): No difference in 100% (1 out of 1) study | SDANN: ↓ in 100% (1 out of 1) studies  SDANN (ln): Did not differ in 100% (1 out of 1) studies | N.A. |
| RMSSD (normalized [nu], logarithmic [ln]) | RMSSD: No difference in 33% (1 out of 3) studies in 3rd trimester  RMSSD: No difference in 67% (2 out of 3) studies in 1st and 2nd trimesters but ↓ in 3rd trimester | RMSSD: ↑ in 20% (1 out of 5) studies, ↓ in 40% (2 out of 5) studies, and no difference in 40% (2 out of 5) studies  RMSSD (ln): No difference in 100% (1 out of 1) studies. | N.A. |

Explanation: Cardiovascular and hemodynamics As shown in the table, as compared to normotensive women, in gestational hypertension, TP (67%), LF (83%), LF (ln) (50%), HF (67%), HF (ln) (100%), LF/HF (60%), SDANN (100%), and RMSSD (67% in first and second trimester) in majority of the studies showed no significant difference. SDNN (67%), RMSSD (67% in third trimester), and TP (ln) (100%), HF (nu) (67%), however, was lower in gestational hypertension as compared with normotensive women in majority of the studies. LF (nu) (100%) and LF/HF (ln) (100%) were higher in gestational hypertension compared with normotensive woman. In preeclamptic women, TP (75%), TP (ln) (50%), LF (50%), and LF (ln) (60%), HF (ln) (80%), SDNN (ln) (50%), SDANN (ln) (100%), and RMSSD (100%) showed no difference as compared with normotensive women. TP (ln) (50%), HF (50%), HF (nu) (100%), SDNN (60%), and SDANN (100%) were lower in preeclamptic compared with normotensive women. LF (nu) (75%), LF/HF (67%), LF/HF (ln) (75%), and SDNN (ln) (50%) were higher in preeclamptic women as compared to normotensive women. In hypertensive pregnancy disorders, TP (100%), LF (100%), LF/HF (100%), and HF (50%) did not differ from normotensive women. Please see Table 4 for more information.
